# Glucocorticoid Receptor Alpha as an Integrative Regulator of Host Defense: A Systems-Level Review and Framework for Immune Regulation and Homeostatic Correction

**DOI:** 10.3390/biomedicines14102313

**Published:** 2026-10-12

**Authors:** Gianfranco Umberto Meduri

**Affiliations:** Department of Medicine and Pharmaceutical Sciences, University of Tennessee Health Science Center, Memphis, TN 38163, USA; gmeduri@uthsc.edu

**Keywords:** glucocorticoid receptor alpha (GRα), host defense, homeostatic correction

## Abstract

Current concepts recognize that effective host defense depends on coordinated interactions among innate and adaptive immunity, inflammatory signaling, immunometabolic regulation, and multiple complementary adaptive biological processes. However, far less attention has focused on the regulatory systems that integrate these diverse responses across the evolving phases of the host response. This Review proposes that glucocorticoid receptor alpha (GRα) functions as a systems-level integrative regulator that may contribute to the integration of immune signaling, antimicrobial competence, mitochondrial bioenergetics, inflammatory regulation, and other adaptive processes across partially overlapping adaptive phases of homeostatic correction. A central premise is that these phase-specific actions are shaped by dynamic changes in chromatin accessibility and transcription-factor interactions, providing a molecular basis for differential GRα regulation throughout the adaptive response. Rather than functioning solely as a transcriptional regulator of inflammation, GRα influences biological processes involved in pathogen sensing, NF-κB/AP-1 signaling, immunometabolic adaptation, innate and adaptive immune responses, endothelial and epithelial barrier integrity, lymphatic function, tissue repair, and the transition from early host defense to inflammatory resolution and restoration of physiological homeostasis. These integrated biological processes are organized within the proposed Priming, Modulatory, and Restorative phases of homeostatic correction. By synthesizing evidence across multiple physiological systems, this Review provides a biologically plausible model for understanding how GRα-associated regulatory mechanisms may contribute to immune effectiveness, disease tolerance, host-defense outcomes, and adaptive recovery during infection and critical illness. The proposed model generates testable hypotheses to guide future mechanistic investigation and the development of precision therapeutic strategies.

## 1. Introduction

Effective host defense is increasingly recognized as a dynamic, systems-level process requiring coordinated interactions among pathogen recognition, innate and adaptive immune responses, inflammatory regulation, resistance mechanisms, disease tolerance, immunometabolic adaptation, and tissue-protective responses [1,2]. Rather than depending solely on efficient pathogen elimination, successful host defense requires an appropriate balance between resistance mechanisms that reduce pathogen burden and disease-tolerance mechanisms that preserve tissue integrity and physiological function during infection [3,4]. More recent studies further emphasize that these coordinated adaptive responses operate as integrated, systems-level biological networks rather than isolated immune pathways [5,6].

Although researchers have studied these adaptive biological processes extensively in isolation, far less attention has focused on the integrative regulatory mechanisms that shape their functional and temporal interactions throughout the host response to infection [5,6]. This Review proposes that glucocorticoid receptor alpha (GRα) functions as a systems-level integrative regulator that contributes to the coordination of these adaptive biological processes across the temporal phases of homeostatic correction, thereby influencing host-defense effectiveness, adaptation to infection, recovery, and restoration of physiological homeostasis [7,8,9]. Designation of GRα as a central integrative regulator does not imply that all components of host defense are GRα-dependent or that GRα exerts uniform or synchronous effects across tissues. Rather, integration emerges from context- and phase-dependent interactions of GC–GRα signaling with interdependent but partially autonomous immune, metabolic, vascular, neuroendocrine, barrier, lymphatic, mitochondrial, and regenerative networks whose adaptive responses may overlap and proceed asynchronously.

The conceptual advance of the present Review does not reside in proposing GRα as a systems-level regulator or in introducing the Priming, Modulatory, and Restorative phases of homeostatic correction, concepts developed in previous work [7,8,9]. Rather, the present Review extends this framework specifically to host defense by integrating pathogen recognition and downstream signaling, antimicrobial competence, innate and adaptive immunity, immunometabolic regulation, resistance and disease tolerance, endothelial and epithelial barrier function, mitochondrial biology, inflammatory resolution, and tissue repair within a unified host-defense-centered framework. This perspective is needed because these processes are commonly examined as separate components of immunity or physiological adaptation, whereas effective host defense depends on their dynamic interaction across tissues and over the course of infection and recovery. The Review therefore examines whether established context-, tissue-, and phase-dependent actions of GC–GR signaling collectively support such integration, without implying that all components of host defense are GRα-dependent or that GRα directly coordinates them through a single causal mechanism. This synthesis generates testable predictions, including whether phase- and tissue-specific alterations in GC–GR signaling are associated with transitions between effective pathogen resistance, disease tolerance, maladaptive host-defense states, and recovery, and whether longitudinal multidomain measurements can ultimately identify these regulatory patterns.

This Review distinguishes between experimentally demonstrated regulatory actions of GRα and the systems-level integration proposed here as a conceptual synthesis. Evidence that GRα regulates or influences individual immune, metabolic, vascular, mitochondrial, barrier, neuroendocrine, or reparative processes does not by itself establish that GRα directly coordinates these processes across tissues or organ systems. Accordingly, terms implying direct causal coordination are used only where supported by the cited evidence; elsewhere, the framework should be understood as integrating established context-specific actions of GC–GR signaling into a testable systems-level model.

### Literature Search and Evidence Synthesis

A structured, hypothesis-driven literature search was conducted from July through September 2026 using PubMed/MEDLINE and Google Scholar, supplemented by reference-list review and Consensus AI-assisted scientific literature searching, evidence discovery, and evidence synthesis. Searches were organized around predefined biological domains relevant to glucocorticoid–GR signaling and host defense; representative domain-specific queries are provided in Table 1. Primary experimental, translational, and clinical studies were prioritized when available, with authoritative reviews used for broader mechanistic and conceptual context. Publications identified through AI-assisted searches were manually verified against the original scientific literature before inclusion. Studies were selected according to their biological and mechanistic relevance to the proposed framework, and evidence supporting, qualifying, or contradicting the framework was considered. No formal language or publication-date restrictions were imposed. Because this is a conceptual narrative Review rather than a systematic or scoping review, formal systematic-review procedures, quantitative evidence grading, and exhaustive study inclusion were not undertaken. Consensus AI was accessed from July through September 2026; a specific platform version was not recorded.

Table 1 [3,4,7,8,10,11,12,13,14,15,16,17,18,19,20,21] summarizes the framework used to guide evidence acquisition, organization, and synthesis throughout this Review and provides a transparent conceptual roadmap for the evidence discussed in the following sections.

This table presents a structured, hypothesis-driven query framework developed to evaluate the role of glucocorticoid receptor alpha (GRα) in immune sensing, host defense, and immune effectiveness. Artificial intelligence (Consensus AI) was used exclusively to generate structured, domain-specific literature queries and facilitate evidence retrieval. The scientific framework, interpretation of the evidence, synthesis of the literature, table organization, and all scientific conclusions were independently developed, critically reviewed, and approved by the author.

Queries are organized across major immune domains, including pathogen recognition, innate immune activation, antimicrobial function, immunometabolism, inflammatory regulation, host defense outcomes, and resolution and repair. These domains are mapped onto the phases of homeostatic correction (Priming, Modulatory, and Restorative), reflecting the temporal evolution and functional organization of immune responses during host defense. Each query tests a specific aspect of GRα-mediated regulation, linking molecular signaling pathways (e.g., PRR activation, NF-κB/AP-1 signaling, mitochondrial function) to functional outcomes such as pathogen clearance, immune effectiveness, and restoration of homeostasis. The table includes representative references for each functional domain to support verification of the evidence base.

This structured approach enables structured evaluation of whether GRα functions as a central integrator of immune sensing, signaling, and coordinated immune responses across different phases of host defense, rather than as a purely anti-inflammatory or immunosuppressive mediator. By integrating temporal phase dynamics with receptor-level signaling, transcriptional control, metabolic regulation, and effector outcomes, the framework provides a basis for evaluating how GRα signaling helps coordinate the transition between pathogen-clearing and pathogen-permissive states. Collectively, the table summarizes the evidence used to evaluate the proposed role of GRα as a systems-level integrator of host defense and phase-specific homeostatic correction.

## 2. Glucocorticoid Receptor Alpha: An Integrative Regulator of Host Defense

GRα is a ligand-activated transcription factor in the nuclear receptor superfamily, expressed in nearly all nucleated cells [22]. Primarily activated by endogenous glucocorticoids, GRα integrates neuroendocrine stress signals with immune, metabolic, vascular, and reparative pathways through tissue-specific, context-dependent gene regulation that varies by cell type, local microenvironment, and the temporal phase of the host response [19,22]. Beyond regulating inflammatory gene transcription, GRα also influences mitochondrial bioenergetics, cellular metabolism, endothelial and epithelial barrier integrity, circadian regulation, oxidative stress, and inter-organ communication, thereby contributing to organism-wide adaptation to physiological stress and infection [7,18]. GRα functions in both the nucleus and mitochondria, providing a mechanism for coordinating nuclear gene expression with mitochondrial bioenergetics and cellular metabolism [16,18,23].

These broad biological actions position GRα as a plausible integrative regulator of the adaptive processes that collectively determine effective host defense. From an evolutionary perspective, this capacity to coordinate diverse adaptive processes supports the hypothesis that GRα functions as a core survival receptor that contributes to organism-wide adaptation during physiological stress, infection, and critical illness [8].

Rather than functioning solely as an anti-inflammatory receptor, GRα participates in the coordinated regulation of pathogen sensing, inflammatory signaling, antimicrobial competence, immunometabolic adaptation, vascular stability, and immune resolution and recovery, in accordance with the host response’s temporal and tissue-specific requirements [12,20,24]. These diverse biological actions are mediated by genomic and non-genomic signaling mechanisms that vary by cell type, tissue microenvironment, and the evolving phase of the host response [19,25]. The context dependence of GRα signaling is further shaped by chromatin architecture, which determines the accessibility of specific regulatory regions and thereby influences which transcriptional programs GRα can engage across different cells and biological states.

Importantly, phase-specific GC–GR responses depend not only on circulating glucocorticoid availability or GRα abundance but also on dynamic changes in receptor sensitivity and competence. These are influenced by GR isoform expression, including GRβ, GRγ, GR-P, and related receptor variants, receptor nuclear translocation, intracellular coregulators, post-translational modifications, tissue-specific glucocorticoid metabolism, chromatin accessibility, and acquired glucocorticoid resistance. GR isoform expression and relative abundance may differ among cell populations and change over the course of critical illness, further contributing to heterogeneity in glucocorticoid responsiveness. Because these determinants vary among cell types, tissues, and over time during severe physiological stress, identical systemic glucocorticoid exposure may generate divergent local responses. These observations argue against interpreting GRα signaling as a uniform organism-wide state. Accordingly, transitions among the proposed Priming, Modulatory, and Restorative programs may reflect changes in tissue-specific GC–GR sensitivity as well as changes in glucocorticoid availability and local GR signaling.

Endogenous glucocorticoid signaling should be distinguished from exogenous corticosteroid administration. Endogenous cortisol secretion is dynamically regulated by the HPA axis in response to physiological stress, whereas pharmacologic administration of hydrocortisone, methylprednisolone, or dexamethasone produces exposures that differ in dose, timing, duration, pharmacokinetics, and tissue distribution. Although both engage GR-dependent pathways, findings derived from pharmacologic corticosteroid administration should not be considered biologically equivalent to endogenous stress-responsive glucocorticoid–GR signaling.

### Chromatin Remodeling Provides the Molecular Basis for Phase-Specific GRα Regulation

The remarkable ability of GRα to coordinate diverse biological functions across multiple tissues cannot be explained solely by ligand binding or receptor abundance. A key determinant of this context-dependent regulation is chromatin—the dynamic organization of DNA and associated proteins that governs access to the genome [26]. Chromatin determines which genes are accessible for transcription and which remain inactive, thereby providing a molecular basis for selective gene regulation during physiological adaptation. Thus, although every nucleated cell contains essentially the same DNA, only selected genes are available for activation at any given time.

Chromatin is not static. During physiological stress and infection, chromatin architecture is continuously remodeled, exposing some regions of DNA while restricting access to others. Importantly, most GRα binding occurs at regulatory regions that are already accessible before glucocorticoid activation, indicating that baseline chromatin accessibility is a major determinant of tissue-specific GRα binding and the resulting transcriptional responses [27,28]. Changes in chromatin accessibility determine which transcription factors—including GRα, NF-κB, and AP-1—can bind specific regulatory regions and regulate gene expression. Consequently, the biological actions of GRα are determined not only by receptor activation but also by the dynamic chromatin landscape in which the receptor operates [24,25,29].

During physiological stress and infection, activation of pattern-recognition receptors rapidly induces NF-κB, AP-1, and other stress-responsive transcription factors, which in turn recruit chromatin-remodeling complexes to thousands of regulatory regions across the genome. This process increases chromatin accessibility, enabling coordinated binding of GRα with other transcriptional regulators. In addition to recognizing accessible chromatin, GRα recruits chromatin-remodeling complexes that further modify local chromatin accessibility, reorganize nucleosomes, and facilitate the recruitment of additional transcriptional regulators, thereby shaping context- and phase-specific transcriptional programs [30,31,32].

Rather than functioning independently, GRα, NF-κB, and AP-1 operate within a shared chromatin environment in which cooperative and antagonistic interactions shape the magnitude, timing, and tissue specificity of gene expression [24,25,33]. As chromatin accessibility changes during the host response, distinct regulatory regions become accessible to transcription-factor binding, enabling GRα to engage in different transcriptional programs as physiological conditions evolve [24,25,29].

This chromatin-based model provides a molecular framework for understanding how GRα’s actions may evolve across the Priming, Modulatory, and Restorative Phases described in this Review. During the Priming Phase, stress-induced chromatin remodeling may facilitate GRα-dependent transcriptional programs that support pathogen recognition, innate immune activation, immunometabolic adaptation, and antimicrobial defense [7,24,25,33]. During the Modulatory and Restorative Phases, evolving chromatin accessibility may enable transcriptional programs that restrain excessive inflammation, preserve endothelial and mitochondrial function, promote tissue repair, and ultimately restore physiologic homeostasis [7,8,29].

**Figure 1:**
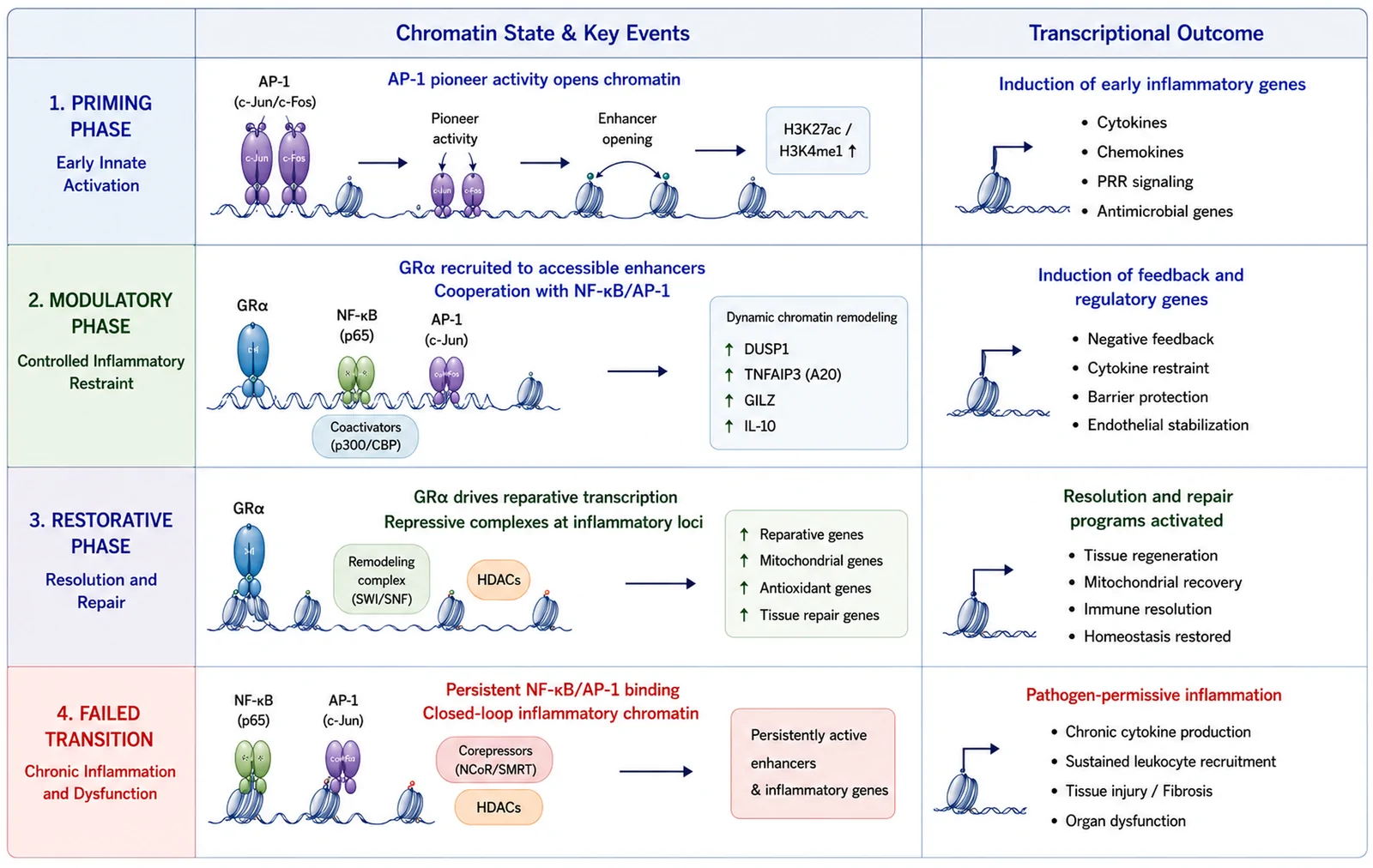
Three Phases of Homeostatic Correction and Phase-Specific GRα Regulation During Severe Physiological Stress. **Abbreviations:** HPA, hypothalamic–pituitary–adrenal; CRH, corticotropin-releasing hormone; ACTH, adrenocorticotropic hormone; GRα, glucocorticoid receptor alpha. **Explanation of colors and arrows:** The color-coded panels distinguish the four proposed response states: light blue, Priming Phase (early innate immune activation); light green, Modulatory Phase (inflammatory restraint and regulatory adaptation); light purple, Restorative Phase (resolution and tissue repair); and light red, Failed Transition (persistent inflammation and dysfunction). Horizontal arrows indicate the progression of chromatin remodeling and associated transcriptional events toward their respective gene-expression outcomes. Curved arrows illustrate local chromatin interactions and enhancer remodeling. Upward arrows (↑) indicate increased histone modifications or gene expression. The differently colored molecular structures distinguish transcription factors and chromatin-associated regulatory components.

Accordingly, dynamic chromatin remodeling provides a plausible mechanism through which the same activated GRα receptor can engage distinct transcriptional programs as the host response progresses from pathogen recognition to inflammatory modulation and ultimately tissue repair and restoration of physiological homeostasis. Thus, the diverse biological functions of GRα reflect, in part, dynamic changes in chromatin accessibility and transcription-factor interactions that may contribute to the phase-specific regulation of homeostatic correction, as summarized in Figure 1 [7,8,9].

For operational purposes, the Priming, Modulatory, and Restorative phases should be regarded as dominant regulatory programs rather than discrete chronological stages with fixed temporal boundaries. Their identification will therefore require longitudinal assessment of multidomain biological trajectories rather than a single biomarker or time point. Candidate measurable features include inflammatory and immune activity, neuroendocrine and GC–GR signaling, metabolic and mitochondrial function, vascular and endothelial integrity, indices of tissue injury, and markers of resolution and repair. The Priming Phase is characterized by increased pathogen- or danger-sensing and innate immune activation, accompanied by mobilization of metabolic and neuroendocrine adaptive responses. The Modulatory Phase is characterized by recalibration of inflammatory amplification, together with preservation of antimicrobial competence and systemic physiological stability. The Restorative Phase is characterized by predominant inflammatory resolution, tissue repair and regeneration, metabolic recovery, and progressive restoration of physiological homeostasis. Because these programs may overlap and progress at different rates across tissues and cellular compartments, transitions between phases should be identified by longitudinal changes showing that the biological features characteristic of one phase are becoming less dominant while those characteristic of another are becoming more prominent, rather than by fixed time points or rigid temporal thresholds.

Legend: This schematic illustrates the proposed framework for glucocorticoid receptor alpha (GRα)-mediated homeostatic correction during severe physiological stress and critical illness. The hypothalamic–pituitary–adrenal (HPA) axis is the principal neuroendocrine stress-response system. Physiological stress and infection stimulate hypothalamic corticotropin-releasing hormone (CRH) release, which induces pituitary adrenocorticotropic hormone (ACTH) secretion and subsequent adrenal glucocorticoid production. Activated glucocorticoids bind GRα, which mediates adaptive responses throughout the three phases of homeostatic correction while simultaneously providing negative feedback to the hypothalamus and pituitary to regulate HPA-axis activity. The adaptive response unfolds in three dynamic, partially overlapping phases: the Priming Phase, the Modulatory Phase, and the Restorative Phase. 

The Priming Phase initiates early host-defense and adaptive-readiness responses, including immune activation, immunometabolic adaptation, mitochondrial activation, barrier defense, cardiovascular adaptation, antioxidant responses, and neuroendocrine stress signaling. The Modulatory Phase calibrates inflammatory amplification and preserves systemic stability by coordinating regulation of inflammatory signaling, endothelial and barrier integrity, vascular homeostasis, redox balance, and integrated immune–metabolic adaptation. The Restorative Phase promotes resolution of inflammation, tissue repair, extracellular matrix remodeling, adaptive immune recalibration, metabolic recovery, and restoration of organ function and physiological resilience. GRα is active throughout all three phases, although its predominant biological functions evolve according to the temporal stage of the host response. Across all phases, GRα functions as a systems-level integrator coordinating immune, metabolic, vascular, neuroendocrine, barrier, and reparative responses during homeostatic correction.

The author conceptually developed this figure with assistance from ChatGPT-6 (OpenAI 2026) for graphical generation, visual organization, and figure refinement. The author independently developed, critically reviewed, and approved the biological framework, scientific content, interpretation, and final figure design.

## 3. Biological Basis for GRα-Mediated Integration of Host Defense

### 3.1. Ubiquitous Cellular Distribution Enables System-Wide Coordination of Host Defense

GRα is expressed in virtually all nucleated cells, including innate and adaptive immune cells, endothelial and epithelial cells, fibroblasts, neurons, hepatocytes, skeletal muscle, adipose tissue, and other parenchymal organs [22]. Unlike most immune regulatory molecules, whose actions are largely confined to specific cell populations or signaling pathways, the nearly ubiquitous distribution of GRα provides a biological basis for regulating adaptive responses across multiple tissues and organ systems, thereby enabling organism-wide integration of the host response [8].

In addition to its well-established nuclear actions, GRα also localizes to mitochondria, where it directly influences mitochondrial gene expression, bioenergetics, oxidative phosphorylation, reactive oxygen species homeostasis, and cellular adaptation to physiological stress [16,34]. This dual nuclear-mitochondrial localization provides a mechanistic basis for coupling nuclear transcriptional programs with mitochondrial energy metabolism, thereby linking immune regulation to the bioenergetic requirements of effective host defense [18].

This broad cellular distribution and dual subcellular localization enable neuroendocrine stress signals to be translated into coordinated transcriptional, metabolic, vascular, immunologic, and reparative responses throughout the organism [7]. Consequently, GRα is well positioned to help coordinate adaptive cellular responses across diverse tissues, thereby integrating immune regulation with metabolic, vascular, mitochondrial, neuroendocrine, and other physiological processes required for effective host defense during infection and other forms of biological stress [8,18].

The following sections examine these major functional domains individually while emphasizing their coordinated integration during homeostatic correction. Table 2 [4,7,8,10,13,14,16,17,19,20,21,35] provides an overview of these interconnected biological programs and summarizes the evidence for GRα regulation of pathogen recognition, innate and adaptive immunity, inflammatory regulation, tissue repair, and restoration of physiological homeostasis across the temporal phases of the host response.

This table summarizes the major functional domains of immune responses and their relevance to glucocorticoid receptor alpha (GRα)-mediated regulation within the framework of homeostatic correction. Immune responses are organized according to their roles in pathogen recognition, innate and adaptive host defense, inflammatory resolution and tissue repair, and long-term adaptation and maintenance of physiological homeostasis.

Pathogen recognition is mediated by pattern-recognition receptors (PRRs), including Toll-like receptors (TLRs), nucleotide-binding oligomerization domain–like receptors (NLRs), and RIG-I–like receptors (RLRs), which detect pathogen-associated molecular patterns (PAMPs) and damage-associated molecular patterns (DAMPs) and activate downstream signaling pathways involving nuclear factor kappa B (NF-κB), activator protein-1 (AP-1), and interferon regulatory factors (IRFs).

Within this integrated system, GRα does not directly mediate pathogen recognition but modulates signaling and transcriptional responses downstream of pathogen recognition. GRα dynamically modulates PRR-driven signaling, cytokine production, immunometabolic adaptation, and downstream effector pathways in a tissue- and phase-specific manner, thereby coordinating immune sensing with antimicrobial effectiveness, tissue protection, and restoration of homeostasis.

Importantly, GRα signaling helps determine whether inflammatory responses remain pathogen-clearing or progress toward pathogen-permissive states. Appropriate GRα signaling may support mitochondrial function, phagolysosomal integrity, intracellular pathogen killing, and coordinated antimicrobial defense while constraining excessive NF-κB- and AP-1-driven inflammatory amplification. In contrast, impaired GRα signaling may contribute to dysregulated inflammatory responses that promote pathogen persistence, tissue injury, immune dysfunction, and impaired host defense.

Accordingly, effective immune regulation requires coordinated GRα-mediated integration of inflammatory control, antimicrobial competence, adaptive immune recalibration, and tissue repair, emphasizing that reduction in inflammatory cytokines alone does not necessarily indicate restoration of immune function or homeostatic resilience. 

### 3.2. GRα Modulates Early Host Defense Following Pathogen Recognition

Host defense begins when pattern-recognition receptors (PRRs), including Toll-like receptors (TLRs), NOD-like receptors (NLRs), RIG-I-like receptors (RLRs), and the cGAS-STING pathway, recognize pathogen-associated molecular patterns (PAMPs) and damage-associated molecular patterns (DAMPs) [10,11]. Activation of these evolutionarily conserved sensing systems triggers intracellular signaling cascades that induce inflammatory cytokines, type I interferons, antimicrobial mediators, and other early defense mechanisms required for rapid pathogen containment, activation of innate immune effector mechanisms, and initiation of adaptive immunity [36].

GRα does not function as a pathogen-recognition receptor; instead, it modulates the magnitude, duration, and biological consequences of PRR signaling by selectively regulating downstream transcriptional programs [12,37]. Following activation by endogenous glucocorticoids released through the hypothalamic–pituitary–adrenal (HPA) axis during physiological stress and infection, GRα interacts with multiple intracellular signaling pathways to shape the quality and coordination of the early host response while preserving the capacity for effective antimicrobial defense [7,13]. As discussed above, these interactions are influenced by the evolving chromatin landscape, which shapes the accessibility of inflammatory and adaptive regulatory elements and contributes to differential transcriptional responses.

Mechanistically, GRα modulates PRR signaling through multiple complementary pathways. These include transrepression of NF-κB- and AP-1-dependent inflammatory gene expression [37], induction of endogenous inhibitory mediators such as glucocorticoid-induced leucine zipper (GILZ) and mitogen-activated protein kinase phosphatase-1 (MKP-1) [38], and dynamic interactions with transcriptional cofactors and chromatin remodeling complexes that collectively regulate inflammatory gene expression [12,13].

Importantly, growing evidence indicates that GRα’s biological effects during early host defense are highly context-dependent [13]. Although GRα generally restrains excessive inflammatory signaling, it may also maintain—or, under selected cellular and inflammatory conditions, enhance—specific innate immune functions required for effective pathogen control [12,13]. Consequently, GRα is better viewed not simply as an anti-inflammatory receptor but as an integrative regulator of early host defense that calibrates pathogen control and limits immune-mediated tissue injury according to the tissue microenvironment and the evolving host response [3,4].

Accordingly, GRα should not be viewed as uniformly enhancing either pathogen resistance or disease tolerance. Rather, GC–GR signaling helps regulate the dynamic balance between these complementary host-defense strategies. Depending on biological context, this regulation may support antimicrobial effector functions and pathogen control, promote tolerance by limiting inflammatory and tissue injury, or, when excessive, prolonged, or biologically mismatched, suppress immune-effector functions and impair pathogen clearance.

### 3.3. GRα Calibrates Innate Immune Effector Functions

Innate immune effector cells constitute the first cellular line of defense following pathogen recognition, rapidly containing invading microorganisms, producing inflammatory mediators, eliminating infected cells, and initiating adaptive immunity [19]. Effective host defense requires not only rapid activation of these effector mechanisms but also precise regulation of their magnitude, duration, and resolution to prevent excessive tissue injury [39,40].

GRα regulates innate immune effector functions in a cell-, tissue-, and context-dependent manner. During the early Priming Phase of host defense, GRα may support innate immune mechanisms downstream of pathogen recognition, inflammatory signaling, and antimicrobial defense [7]. Early activation of NF-κB and AP-1 also remodels chromatin, creating accessible regulatory regions that influence subsequent GRα-dependent transcriptional responses. As Modulatory and Restorative programs become increasingly expressed—while potentially overlapping with persistent Priming activity in other tissues or cellular compartments, GRα helps recalibrate these responses by limiting excessive inflammation, promoting disease tolerance, and facilitating inflammatory resolution [13]. Through this adaptive recalibration, GRα influences cytokine production, cellular activation, antimicrobial activity, metabolic adaptation, and inflammatory resolution, thereby helping preserve pathogen control while limiting immune-mediated tissue injury [7,12].

Macrophages provide one of the clearest examples of glucocorticoid–GR-mediated regulation during innate host defense. Rather than simply suppressing macrophage function, glucocorticoid– GR signaling influences macrophage activation states, phagocytosis, cytokine production, and antimicrobial defense in ways that may support effective host defense while limiting excessive inflammatory tissue injury [15,41]. These activation states are multidimensional and context-dependent and should not be interpreted as a binary or obligatory sequential M1-to-M2 polarization switch. Recent evidence demonstrates that endogenous glucocorticoid signaling is required for appropriate macrophage activation during *Helicobacter pylori* infection, where loss of GR signaling results in aberrant chromatin remodeling, impaired induction of inflammatory genes, reduced T-cell recruitment, and diminished bacterial control [14].

Natural killer (NK) cells provide a complementary example of GR-mediated immune regulation. During viral infection, endogenous glucocorticoids induce tissue-specific PD-1 expression on splenic NK cells, limiting excessive interferon-γ production and preventing immunopathology without compromising viral clearance [20]. These findings illustrate how glucocorticoid–GR signaling can calibrate immune-effector responses to preserve pathogen control while limiting collateral tissue injury [40].

Neutrophils provide another example of glucocorticoid–GR-mediated regulation during innate host defense. Glucocorticoid–GR signaling can restrain excessive neutrophil recruitment and activation by suppressing endothelial adhesion, chemotactic signaling, NF-κB activation, and reactive oxygen species generation, thereby reducing collateral tissue injury [42,43]. However, its effects on phagocytosis, microbial killing, and neutrophil extracellular trap (NET) formation vary according to pathogen, inflammatory milieu, and the timing, dose, and duration of glucocorticoid exposure [44,45]. These observations indicate that glucocorticoid–GR signaling can exert context-dependent effects on neutrophil effector functions rather than uniformly suppressing them.

Collectively, these observations support a broader role for glucocorticoid–GR signaling in regulating innate immune effector functions and influencing the subsequent development of adaptive immunity [7,19].Because innate immune responses shape the quality, magnitude, and subsequent regulation of adaptive immune responses, glucocorticoid–GR-mediated regulation of dendritic-cell function and antigen presentation may provide an important mechanistic link between innate and adaptive host defense.

### 3.4. GRα Regulates the Transition from Innate to Adaptive Immunity

Dendritic cells, specialized antigen-presenting cells that capture, process, and present microbial antigens to naïve T lymphocytes, occupy a pivotal position at the interface between innate and adaptive immunity by linking pathogen recognition to antigen presentation and T-cell activation [46,47]. Consequently, regulation of dendritic cell maturation and function provides an important mechanism through which GRα can influence the transition from innate to adaptive immune responses [48].

GRα regulates dendritic-cell maturation and function throughout the evolving host response. Within the proposed framework, during the Priming Phase of host defense, dendritic cells preserve their capacity for pathogen sensing, antigen uptake, processing, migration to secondary lymphoid organs, and initiation of adaptive immune responses [19]. As the host response evolves toward modulation, GRα can recalibrate dendritic-cell function by limiting excessive maturation, reducing co-stimulatory molecule expression, modulating pro-inflammatory cytokine production, and restraining naïve T-lymphocyte activation, thereby promoting immune regulation and limiting excessive adaptive immune activation [49,50]. Rather than uniformly suppressing dendritic-cell function, GRα can modulate the magnitude, quality, and duration of adaptive immune activation according to biological context and the evolving host response [19,48].

#### 3.4.1. GRα Calibrates Adaptive T-Cell Responses

Adaptive T-cell responses are essential for pathogen clearance, immunologic memory, and long-term protection [51]. However, excessive or dysregulated T-cell activation can also be a major source of immune-mediated tissue injury [19]. Consequently, effective host defense requires not only robust adaptive immunity but also precise regulation of T-cell activation, differentiation, trafficking, and resolution [19,51].

GRα regulates adaptive T-cell responses in a cell-, tissue-, and context-dependent manner [19,51]. Rather than uniformly suppressing T-cell immunity, GRα modulates T-cell activation and differentiation according to the biological context, limiting excessive inflammatory responses while supporting effective host defense [51]. Experimental studies show that loss of endogenous GR signaling results in exaggerated T-cell activation, excessive inflammatory cytokine production, and immune-mediated tissue injury, underscoring GRα’s important role in maintaining adaptive immune homeostasis during infection [40].

GRα also shapes the functional specialization and long-term memory of adaptive immunity by regulating helper T-cell differentiation, cytotoxic T-cell responses, and memory-cell development [51]. These effects depend on the T-cell activation state, local inflammatory signals, the tissue microenvironment, and circadian neuroendocrine regulation [52,53]. Consequently, GRα functions not merely as an inhibitor of adaptive immunity but as a regulator that helps balance effective antimicrobial defense with limitation of immune-mediated tissue injury [19].

In addition to regulating effector T-cell activation and differentiation, GRα contributes to adaptive immune homeostasis by stabilizing regulatory T-cell (Treg) function and promoting immune tolerance during inflammation and recovery [54,55]. Experimental studies demonstrate that endogenous GRα signaling preserves the suppressive phenotype of Tregs during inflammation, thereby preventing excessive effector T-cell activation and limiting immune-mediated tissue injury [54,55]. Beyond its direct effects on Tregs, GRα also contributes to inflammatory resolution by regulating pro-resolving programs in macrophages, dendritic cells, and other immune cells, thereby facilitating restoration of immune homeostasis while preserving protective immunity [7,48]. Collectively, these findings further support the concept that GRα functions not as a generalized immunosuppressive receptor but as an adaptive regulator that helps calibrate immune responses to the evolving temporal and biological requirements of host defense [7].

The regulation of cytotoxic CD8^+^ T cells further illustrates GRα’s regulatory breadth in adaptive immunity. During infection, GRα modulates CD8^+^ T-cell activation, differentiation, and effector function, thereby contributing to the balance between antimicrobial immunity and limitation of immune-mediated tissue injury [40,51]. These effects vary according to activation state, inflammatory context, and glucocorticoid exposure. In addition, GRα influences the balance between terminal effector differentiation and memory-cell formation, thereby supporting the development of long-lived protective immunity under appropriate conditions [56]. Together, these observations illustrate how GRα can calibrate cytotoxic T-cell responses while contributing to durable protective immunity [51,56].

#### 3.4.2. GRα Regulates Humoral Immune Responses

B lymphocytes are the principal effector cells of humoral immunity, differentiating into antibody-producing plasma cells and memory B cells that confer long-term protection against recurrent infection [57]. Although GRα’s effects on B-cell biology have been studied less extensively than its actions on innate immune cells and T cells, accumulating evidence indicates that GRα regulates B-cell activation, survival, trafficking, differentiation, antibody production, and memory-cell development in a context-dependent manner [19,58].

Rather than functioning solely as a suppressor of antibody production, endogenous GRα signaling modulates B-cell receptor (BCR) and Toll-like receptor (TLR) signaling, regulates CXCR4-dependent trafficking, and contributes to specific antibody responses while maintaining immune homeostasis [40,57]. GRα also influences the balance between effective humoral immunity and immune regulation by modulating B-cell activation in response to the inflammatory microenvironment and the evolving host response. Collectively, these observations support a broader role for GRα in regulating humoral immunity and in linking B-cell responses to broader adaptive immune processes involved in host defense and immune homeostasis [7,19].

## 4. GRα Coordinates Systemic Adaptive Responses

### 4.1. GRα Preserves Endothelial Function and Microvascular Homeostasis During Host Defense

The vascular endothelium, a dynamic monolayer of specialized cells lining the entire circulatory system, plays a central role in host defense by regulating vascular permeability, leukocyte trafficking, coagulation, vascular tone, and tissue perfusion [59]. During infection, coordinated endothelial responses are essential for delivering immune cells and antimicrobial mediators to sites of injury while preserving microvascular integrity and organ perfusion [60]. Conversely, endothelial dysfunction contributes to capillary leak, tissue edema, dysregulated inflammation, microvascular thrombosis, impaired oxygen delivery, and multiple-organ dysfunction, making preservation of endothelial homeostasis a critical determinant of clinical outcome [18].

GRα is increasingly recognized as a key regulator of endothelial adaptation during infection [7,59] modulating inflammatory signaling, barrier integrity, leukocyte-endothelial interactions, vascular permeability, and microvascular homeostasis [7,8]. Experimental studies demonstrate that endothelial-specific deletion of GR markedly increases susceptibility to endotoxemia and sepsis, providing direct evidence that endothelial GR signaling contributes to vascular stability and survival during severe inflammatory stress [60].

At the cellular level, GRα supports endothelial barrier integrity through multiple complementary mechanisms. GRα suppresses NF-κB-dependent inflammatory activation and supports the expression and organization of adherens and tight junction proteins—including vascular endothelial (VE)-cadherin, occludin, claudin-5, and zonula occludens-1 (ZO-1) [59,61]. GRα also helps preserve endothelial barrier function through mechanisms involving the endothelial glycocalyx and barrier-stabilizing pathways, including sphingosine kinase-1 (SphK1), sphingosine-1-phosphate (S1P), and angiopoietin-1 (Ang-1) [18]. Collectively, these mechanisms help reduce vascular permeability, limit capillary leakage, and preserve tissue oxygenation during systemic inflammation [18,59].

Beyond maintaining barrier integrity, GRα also regulates leukocyte trafficking by regulating endothelial activation rather than preventing immune-cell recruitment [59]. Through suppression of endothelial adhesion molecules, inflammatory cytokines, and chemokine expression, GRα limits excessive leukocyte adhesion and endothelial injury while preserving the controlled recruitment of immune cells required for effective antimicrobial defense [60]. In parallel, GRα contributes to maintenance of vascular tone, nitric oxide homeostasis, tissue perfusion, and microvascular blood flow, thereby linking endothelial regulation to broader systemic adaptation during host response to infection [7,62].

Collectively, these endothelial effects may contribute to preservation of vascular integrity while limiting capillary leak, microvascular dysfunction, and progression to multiple-organ dysfunction during severe infection [18].

### 4.2. GRα Coordinates Immunometabolic Adaptation and Mitochondrial Bioenergetics During Host Defense

Effective host defense is an energetically demanding process that requires coordinated regulation of cellular metabolism to sustain immune activation, antimicrobial defense, tissue repair, and the restoration of physiological homeostasis. Increasing evidence indicates that GRα links neuroendocrine stress responses with immunometabolic adaptation and mitochondrial bioenergetics, thereby contributing to the regulation of metabolic requirements during host defense across multiple tissues and organ systems [7,8,29].

During acute infection, endogenous glucocorticoid–GR signaling contributes to the redistribution of metabolic substrates required to support the increased energetic demands of host defense. Through effects on hepatic gluconeogenesis, lipolysis, proteolysis, and ketogenesis, glucocorticoid–GR signaling contributes to maintaining glucose availability while providing alternative energy substrates for immune cells and metabolically active tissues during physiological stress [29,63]. These systemic metabolic adaptations help provide the energy substrates required to sustain immune and organ function during the evolving host response.

Beyond its well-established nuclear transcriptional actions, evidence from experimental studies indicates that glucocorticoid receptor signaling also occurs within mitochondria [16,23]. Within mitochondria, GR has been reported to interact with mitochondrial DNA and mitochondrial transcriptional machinery, providing evidence for receptor-associated regulation of mitochondrial gene expression; however, the strength and mechanistic resolution of this evidence vary among experimental systems [16,23]. Additional studies have linked glucocorticoid–GR signaling to changes in mitochondrial bioenergetics, including oxidative phosphorylation, ATP production, reactive oxygen species balance, and mitochondrial quality-control processes [16,18,64]. However, these observations derive from different experimental systems and do not establish that all of these effects constitute a single GRα-dependent causal pathway. Moreover, evidence directly demonstrating this complete nuclear–mitochondrial regulatory sequence during infection or host defense remains limited. These findings therefore support a potential role for mitochondrial glucocorticoid–GR signaling in coupling cellular stress responses to bioenergetic adaptation, while the extent and context dependence of this contribution during host defense require further investigation.

GRα also regulates immunometabolic adaptation within immune cells. During the Priming Phase of host defense, immune cells undergo metabolic reprogramming to support rapid antimicrobial activity and inflammatory signaling. As the host response evolves toward modulation and recovery, GRα contributes to metabolic reprogramming from predominantly inflammatory glycolytic programs toward greater reliance on mitochondrial oxidative phosphorylation and tricarboxylic acid (TCA)-cycle activity, thereby limiting excessive inflammation and supporting disease tolerance, inflammatory resolution, and tissue repair [17,65,66]. In macrophages, these metabolic adaptations are essential for optimal glucocorticoid responsiveness and directly regulate inflammatory gene expression and antimicrobial defense [17,65].

Together, these metabolic effects may help align energy availability with the changing demands of host defense, inflammatory resolution, tissue repair, and recovery [7,8,18].

### 4.3. GRα Regulates Intestinal Barrier Integrity and Microbiome–Host Interactions During Host Defense

The intestinal barrier is a dynamic interface composed of epithelial cells, tight junctions, mucus, resident immune cells, and the gut microbiota, which collectively restrict microbial translocation while permitting nutrient absorption and regulated communication between the host and its microbial environment [67,68]. During infection and critical illness, disruption of this barrier can promote dysbiosis, increased intestinal permeability, bacterial and endotoxin translocation, systemic inflammation, and remote-organ injury [18,68].

GRα regulates intestinal barrier integrity through its effects on epithelial inflammatory signaling, tight-junction integrity, mucosal renewal, and local immune response [18,69]. Intestinal GR signaling restrains STAT1- and TNF-dependent inflammatory injury, thereby helping preserve epithelial integrity during severe inflammation [70]. However, its effects are not uniformly barrier protective. In experimental models combining glucocorticoid exposure with ethanol-induced epithelial injury, intestinal epithelial GR was associated with tight-junction disruption, increased permeability, endotoxemia, and systemic inflammation, showing that GRα effects on barrier function vary by biological context and the nature of the associated tissue injury [71].

GRα also participates in bidirectional communication among the intestinal epithelium, the gut microbiota, and the hypothalamic–pituitary–adrenal (HPA) axis. Glucocorticoid signaling can alter microbial composition. Conversely, microbial signals and metabolites—including short-chain fatty acids, bile-acid derivatives, and other immunometabolites—can influence neuroendocrine activity, local glucocorticoid availability, and downstream GR signaling [18,72,73]. Experimental studies further indicate that glucocorticoid-associated alterations in microbial communities can have divergent effects, including dysbiosis or modulation of inflammatory responses, depending on treatment duration, epithelial integrity, and the underlying inflammatory environment [71,74].

Through these reciprocal interactions, GRα integrates epithelial barrier integrity, microbiome–host communication, mucosal immunity, immunometabolic adaptation, and systemic host defense [18,69]. Appropriate GRα signaling may help limit microbial translocation, systemic inflammatory amplification, and remote-organ injury while supporting epithelial repair and restoration of intestinal homeostasis. Conversely, excessive, prolonged, or biologically mismatched glucocorticoid signaling may disrupt epithelial and microbial equilibrium.

Barrier disruption may also create a self-amplifying interaction among microbial exposure, inflammation, and impaired host defense. Experimental studies demonstrate GR-dependent regulation of epithelial barrier integrity in both intestinal and respiratory tissues, whereas excessive or contextually inappropriate glucocorticoid signaling can alter microbial communities and antimicrobial defenses [71,75,76,77]. Once barrier integrity is compromised, microbial products or organisms may gain greater access to host tissues, amplifying inflammatory signaling and systemic exposure. Importantly, this interaction may be bidirectional: elevated concentrations of pro-inflammatory cytokines, including IL-1β, IL-6, and TNF-α, can enhance both extracellular and intracellular bacterial growth, suggesting that dysregulated inflammation may not only damage host tissues but also create conditions that favor microbial proliferation and persistence [78,79,80].

Consistent with this reciprocal host–microbe interaction, recent multi-omics analysis of patients with acute, severe lower respiratory tract infection identified distinct lower-respiratory microbial community states associated with divergent local immune responses and clinical outcomes, including pathogen-dominant states linked to heightened inflammatory signaling and poor prognosis [81]. In parallel, dysregulated inflammatory and metabolic responses may impair effective antimicrobial function and pathogen clearance. Although these observations do not establish a single GRα-dependent pathway in humans, they are consistent with a context-dependent contribution of glucocorticoid–GR signaling to mucosal barrier function and host–microbiome interactions [18,82].

## 5. GRα Integrates Organ-System Communication During Homeostatic Correction

### 5.1. GRα Facilitates Lymphatic Function and Inflammatory Resolution During Host Defense

The lymphatic system is an essential component of host defense, serving as the principal conduit for tissue-fluid drainage, antigen transport, immune-cell trafficking, and communication between peripheral tissues and secondary lymphoid organs. Efficient lymphatic function promotes clearance of excess interstitial fluid, inflammatory mediators, microbial products, and cellular debris while facilitating dendritic-cell migration and adaptive immune activation. Impaired lymphatic function contributes to persistent tissue edema, prolonged inflammation, impaired immune surveillance, and delayed restoration of tissue homeostasis [83,84].

Experimental evidence indicates that GR signaling directly regulates lymphatic endothelial barrier function. In inflamed airway lymphatics, dexamethasone reversed inflammation-induced remodeling of lymphatic endothelial junctions and restored button-like junctions. Dexamethasone also increased phosphorylated nuclear GR in lymphatic endothelial cells and promoted button formation in the absence of inflammation, supporting a direct receptor-mediated effect on lymphatic endothelial plasticity [85]. In primary lymphatic endothelial cells, glucocorticoid exposure altered junctional gene expression and increased barrier integrity, effects reversed by GR antagonism, supporting receptor-dependent regulation of lymphatic endothelial permeability [18,86]. Additional evidence links glucocorticoid–GR signaling to processes relevant to lymphatic function, including endothelial inflammatory signaling, vascular barrier integrity, leukocyte–endothelial interactions, and tissue-fluid homeostasis [60,87,88]. However, GC–GR effects on lymphatic function are not uniformly protective, as glucocorticoid exposure has also been associated with reduced lymphatic endothelial proliferation and migration and impaired lymphatic vessel contractility [86,89].

By preserving endothelial barrier integrity, suppressing excessive NF-κB-dependent inflammatory activation, limiting leukocyte-endothelial interactions, and maintaining tissue perfusion, glucocorticoid–GR signaling may create physiological conditions that support efficient lymphatic drainage and interstitial fluid homeostasis [59,60].

GRα also contributes indirectly to lymphatic immune function by regulating macrophage activation, dendritic-cell maturation, antigen presentation, and leukocyte trafficking, all of which influence antigen delivery to draining lymph nodes and the initiation and resolution of adaptive immune responses [19,40,48]. During the Modulatory and Restorative Phases, these processes may contribute to debris clearance and the restoration of tissue homeostasis [7,8].

Collectively, available evidence suggests both direct and indirect contributions of glucocorticoid–GR signaling to lymphatic endothelial function and interstitial fluid homeostasis.

### 5.2. GRα Coordinates Neuroendocrine Regulation of Host Defense

Host defense is regulated not only by local immune responses but also by integrated neuroendocrine networks that contribute to systemic adaptation during infection. These networks include the hypothalamic–pituitary–adrenal (HPA) axis, autonomic nervous system, circadian regulatory pathways, and bidirectional neuroimmune communication [19,90]. Consistent with this systems-level interpretation, a recent independent model of sepsis proposes that HPA-axis and glucocorticoid signaling participate in centrally coordinated neuroendocrine–metabolic regulation of the immune response, emphasizing differential effects on innate and adaptive immunity rather than uniform immunosuppression [91]. GRα serves as the principal intracellular effector of glucocorticoid signaling and contributes to tissue-specific transcriptional responses within these neuroendocrine networks [7,18].

Direct experimental evidence demonstrates that GR signaling is required for effective neuroendocrine feedback and host-defense regulation. Genetic disruption of GR impairs negative feedback within the stress axis and reduces survival during acute stress, while cell-specific GR deletion alters susceptibility to endotoxemia and viral infection by disrupting control of IFN-γ, IL-10-dependent tolerance, and PD-1-mediated restraint of immunopathology [20,92,93]. These findings indicate that effective neuroendocrine regulation depends on receptor competence rather than circulating glucocorticoid concentrations alone. Consistent with this concept, human and translational studies of critical illness demonstrate marked tissue- and cell-specific differences in GRα expression and signaling despite shared systemic glucocorticoid exposure, with suppression in circulating neutrophils but preservation or enhancement of signaling in other tissues and immune-cell populations [94,95,96].

During bacterial and viral infections, activation of the HPA axis increases endogenous glucocorticoid production in response to inflammatory cytokines and physiological stress [90]. Following activation by endogenous glucocorticoids, glucocorticoid–GR signaling contributes to phase- and tissue-specific transcriptional programs that may support early innate immune and metabolic adaptation, subsequently limit excessive inflammatory injury, and participate in tissue repair and restoration of physiological homeostasis [7,18]. Intact GRα signaling is equally important for negative feedback regulation of the HPA axis [90], whereas impaired receptor abundance or function may contribute to reduced glucocorticoid responsiveness during critical illness and represents one component of the broader pathophysiology associated with critical illness-related corticosteroid insufficiency (CIRCI) [7].

GRα also interfaces neuroendocrine regulation with autonomic and circadian control of immunity. Circadian glucocorticoid secretion coordinates daily oscillations in leukocyte trafficking, cytokine production, lymphocyte maintenance, and immune responsiveness [19,97]. Autonomic neural pathways operate in parallel with and interact with HPA-axis/glucocorticoid signaling in regulating the timing and magnitude of host-defense responses [19]. These interacting neuroendocrine mechanisms contribute to temporal regulation of immune responsiveness in relation to physiological and environmental signals [18,19].

Neuroendocrine regulation of host defense operates through continuous bidirectional communication between the immune and nervous systems [90]. Peripheral inflammatory signals activate central neuroendocrine pathways, whereas glucocorticoid feedback through GRα recalibrates immune and tissue responses as physiological conditions evolve [90]. Conversely, chronic inflammation, hypoxia, altered receptor isoform balance, or glucocorticoid resistance can disrupt GRα signaling, impairing this adaptive neuroimmune communication and contributing to dysregulated host responses [7,98].

### 5.3. GRα Coordinates Tissue Repair and Restoration of Organ Function During Host Defense

Successful host defense extends beyond pathogen elimination to include restoration of tissue integrity, recovery of organ function, and re-establishment of physiological homeostasis. As the initial infectious insult is brought under control, injured tissues must transition from inflammatory defense to coordinated programs of inflammatory resolution, cellular regeneration, extracellular matrix remodeling, angiogenesis, and functional recovery. Glucocorticoid–GR signaling contributes to this transition through effects on immune, stromal, vascular, metabolic, and reparative processes [7,8,18].

One of the principal mechanisms through which GRα contributes to tissue repair is by facilitating the transition from inflammatory effector responses toward pro-resolving immune programs. Rather than simply suppressing inflammation, GR signaling promotes macrophage reprogramming toward reparative phenotypes and induces endogenous pro-resolving mediators, including glucocorticoid-induced leucine zipper (GILZ) and Annexin A1, thereby facilitating inflammatory resolution and tissue repair [7,99]. Direct genetic evidence supports a critical role for macrophage GR in this transition: myeloid GR deletion following myocardial infarction disrupts macrophage reparative programming, impairs scar formation and angiogenesis, worsens ventricular remodeling and cardiac function, and increases mortality from cardiac rupture [100]. Experimental studies further demonstrate that glucocorticoid-induced macrophage reprogramming requires AMPKα1-FOXO3 signaling, linking GR-dependent transcriptional responses to metabolic programs required for regenerative inflammation [21].

GRα effects on tissue repair are not uniformly regenerative and vary by cell type, tissue context, and injury phase. Endothelial GR loss accelerates fibrotic remodeling and disrupts metabolic and inflammatory programs, supporting a protective role for endothelial GR during tissue repair. Ref. [101] Conversely, glucocorticoid–GR signaling can restrain selected regenerative processes: glucocorticoid exposure improves epithelial barrier organization while delaying epithelial migration and wound closure, and myeloid GR deletion after spinal cord injury has been associated with improved neurological recovery [102,103]. These divergent effects emphasize that GRα regulates the balance among inflammatory resolution, structural stabilization, remodeling, and regeneration rather than functioning as a uniformly pro-reparative signal.

GR signaling also influences structural repair through effects on immune cells, fibroblasts, endothelial cells, epithelial cells, and the extracellular matrix. Direct receptor-specific studies demonstrate that GR signaling regulates multiple components of post-injury remodeling and regeneration, including scar formation, angiogenesis, fibroblast differentiation, cellular proliferation, and functional recovery. Importantly, these effects are strongly cell- and context-dependent: macrophage GR is required for effective scar formation, angiogenesis, and cardiac remodeling after myocardial infarction, whereas cardiomyocyte GR restrains proliferative regeneration, with GR deletion or antagonism enhancing cardiomyocyte cell-cycle re-entry and cardiac muscle regeneration [100,104,105]. These divergent effects indicate that GRα regulates the balance among inflammatory resolution, structural repair, and regenerative growth rather than functioning as a uniformly pro- or anti-regenerative signal.

Experimental and translational studies further indicate that preserving tissue-specific GRα signaling supports effective repair responses, whereas impaired GRα expression or function may delay recovery and contribute to persistent organ dysfunction during prolonged critical illness [100,106].

These reparative processes are consistent with the biological program proposed for the Restorative Phase of homeostatic correction [7,8,18].

### 5.4. GRα Integrates Inter-Organ Communication Networks During Host Defense

Host defense is increasingly recognized as a systems-level adaptive process that requires coordinated communication among immune, neuroendocrine, metabolic, vascular, lymphatic, epithelial, and stromal tissues, rather than the independent function of individual organ systems. The widespread cellular distribution of GR provides a mechanism through which systemic glucocorticoid signals can generate tissue-specific transcriptional responses according to local biological context [13,19,29].

Tissue-specific responses to systemic glucocorticoid signaling arise in part because glucocorticoid signals are interpreted differently within individual tissues shaped by local chromatin accessibility, GR isoform expression, intracellular coregulators, glucocorticoid metabolism, and the prevailing inflammatory microenvironment [29,98,107]. Emerging evidence further indicates that, in addition to regulation mediated by local chromatin accessibility, GRα also regulates gene expression through long-range three-dimensional chromatin interactions that coordinate enhancer-promoter communication across the genome, providing an additional mechanism by which tissue-specific transcriptional programs are integrated during homeostatic correction [108,109]. These determinants provide a mechanistic basis for divergent tissue responses to a shared systemic glucocorticoid signal [13,106].

## 6. Unified Framework for GRα-Mediated Homeostatic Correction

### 6.1. GRα Coordinates Pathogen Control and Disease Tolerance During Host Defense

Effective host defense requires a dynamic balance between pathogen resistance and disease tolerance. GR signaling may influence this balance by modulating antimicrobial immune functions while limiting immune-mediated tissue injury and preserving physiological function [14,20].

The effects of glucocorticoid–GR signaling on host resistance and tolerance vary with biological context, pathogen, tissue, and timing of the host response. During the early stages of bacterial and viral infections, endogenous glucocorticoid signaling supports appropriate activation of innate immune defenses while mobilizing the metabolic resources required for effective antimicrobial responses [8,12]. As the host response progresses through the Modulatory and Restorative Phases, glucocorticoid–GR signaling may increasingly restrain excessive inflammatory signaling through effects on NF-κB, AP-1, inflammatory cytokines, oxidative stress, and immune cell activation, thereby limiting immunopathology without necessarily compromising pathogen clearance [19,110].

Experimental studies illustrate how endogenous glucocorticoid–GR signaling can influence the balance between pathogen control and disease tolerance [14,20]. During viral infection, glucocorticoid-dependent GRα signaling induces tissue-specific PD-1 expression on splenic natural killer cells, limiting excessive interferon-γ production and preventing immune-mediated tissue injury without impairing viral clearance [20]. Similarly, studies of bacterial infection demonstrate that endogenous GR signaling contributes to appropriate macrophage activation, chromatin remodeling, inflammatory gene regulation, and effective antimicrobial defense, whereas loss of GR signaling results in dysregulated inflammation and impaired control of bacteria [14,111].

The relative contributions of host resistance and disease tolerance are highly context- and phase-dependent. Physiological endogenous glucocorticoid signaling may contribute to adaptive responses that balance pathogen control with tissue fucntion, while appropriately timed therapeutic glucocorticoid administration may support these adaptive processes in selected clinical contexts. In contrast, prolonged, excessive, or biologically inappropriate glucocorticoid exposure may impair host defense and increase susceptibility to secondary infection. Likewise, impaired GRα expression or function, altered receptor isoform balance, chronic inflammation, oxidative stress, and glucocorticoid resistance disrupt this adaptive equilibrium, contributing to persistent inflammation, tissue injury, and organ dysfunction [19,98,112].

### 6.2. Integrated Phase-Specific Framework for Homeostatic Correction

**Figure 2:**
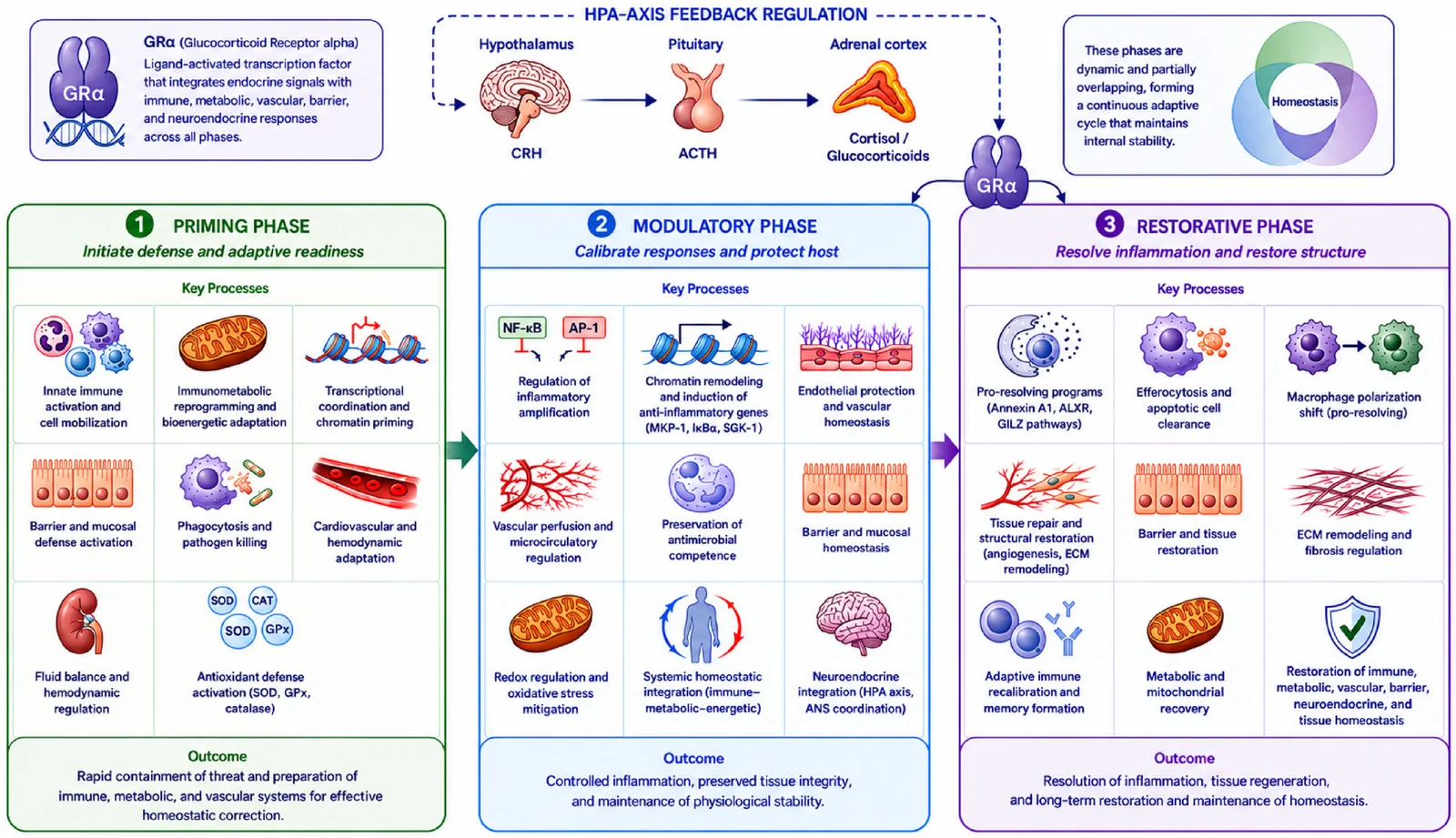
Phase-Specific GRα Regulatory Programs Coordinate Successful and Failed Homeostatic Correction. **Abbreviations:** GRα, glucocorticoid receptor alpha; PAMPs, pathogen-associated molecular patterns; DAMPs, damage-associated molecular patterns; PRR, pattern-recognition receptor; NF-κB, nuclear factor kappa B; AP-1, activator protein-1; TNF, tumor necrosis factor; IL, interleukin; DUSP1, dual-specificity phosphatase 1 (MAPK phosphatase-1); TNFAIP3 (A20), tumor necrosis factor alpha-induced protein 3; GILZ, glucocorticoid-induced leucine zipper; ROS, reactive oxygen species; ATP, adenosine triphosphate; PPP, pentose phosphate pathway; OXPHOS, oxidative phosphorylation; Nrf2, nuclear factor erythroid 2-related factor 2; mTOR, mechanistic target of rapamycin; TF, transcription factor; H3K27ac, histone H3 lysine 27 acetylation; H3K4me1, histone H3 lysine 4 monomethylation; CBP, CREB-binding protein; ECM, extracellular matrix; ALXR, lipoxin A4 receptor; TGF-β, transforming growth factor-beta; VEGF, vascular endothelial growth factor; KGF, keratinocyte growth factor. **Color coding:** Green, blue, and purple identify the Priming, Modulatory, and Restorative phases, respectively. The corresponding arrows indicate progression between overlapping adaptive phases rather than discrete or strictly sequential transitions. Colors are used for visual organization and do not represent quantitative differences in biological activity.

The evidence reviewed above provides the basis for a conceptual framework in which glucocorticoid–GR signaling contributes to distinct but interacting biological programs during homeostatic correction. The adaptive response unfolds in three dynamic, partially overlapping phases: the Priming Phase, the Modulatory Phase, and the Restorative Phase (Figure 2).

This figure illustrates the proposed mechanistic framework by which glucocorticoid receptor alpha (GRα) may contribute to the regulation of inflammatory signaling, metabolic adaptation, chromatin remodeling, immune regulation, and tissue repair across the temporal phases of homeostatic correction. The central GRα row summarizes the evolving phase-specific regulatory programs of GRα in relation to the inflammatory, metabolic, transcriptional, vascular, barrier-protective, and reparative processes illustrated in the surrounding functional domains. GRα progressively shifts from facilitating adaptive host-defense responses during the Priming Phase to calibrating inflammatory and metabolic homeostasis during the Modulatory Phase, and finally to promoting reparative gene programs and restoring physiological homeostasis during the Restorative Phase.

The three left panels depict successful adaptive progression through the Priming, Modulatory, and Restorative phases, whereas the right panel illustrates failed transition states associated with dysregulated GRα signaling, glucocorticoid resistance, persistent inflammatory activation, metabolic dysfunction, impaired reparative programming, and pathogen-permissive inflammation.

During the Priming Phase, GRα is proposed to contribute to responses downstream of pathogen recognition, leukocyte recruitment, inflammatory activation, mitochondrial adaptation, glycolytic energy mobilization, chromatin accessibility, and antimicrobial competence required for early host defense. During the Modulatory Phase, GRα is proposed to contribute to the regulation of NF-κB/AP-1 signaling, chromatin remodeling, endothelial stabilization, mitochondrial preservation, metabolic flexibility, and the induction of negative-feedback and tissue-protective pathways. As homeostatic correction progresses into the Restorative Phase, GRα may contribute to reparative transcriptional reprogramming, oxidative metabolism, mitochondrial recovery, efferocytosis, tissue repair, extracellular matrix remodeling, and restoration of immune and physiological homeostasis.

The failed-transition pathway illustrates how impaired or dysregulated GRα signaling may disrupt the coordinated progression toward resolution, resulting in persistent NF-κB/AP-1 activation, inflammasome amplification, mitochondrial dysfunction, defective chromatin remodeling, impaired reparative responses, tissue injury, and chronic pathogen-permissive inflammatory states. This panel illustrates the hypothesis that failure of homeostatic correction may involve disruption of phase-specific glucocorticoid–GR responses rather than alteration of a single inflammatory pathway.

The author conceptually developed this figure with assistance from ChatGPT (OpenAI) for graphical generation, visual organization, and figure refinement. The author independently developed, critically reviewed, and approved the biological framework, scientific content, interpretation, and final figure design.

Table 3 [4,7,8,10,13,14,16,17,19,20,21,35] integrates these concepts by synthesizing the principal adaptive biological programs within the Priming, Modulatory, and Restorative Phases of homeostatic correction, providing a systems-level overview of the coordinated biological processes discussed throughout this Review.

Table 3 summarizes the principal glucorticoid receptor alpha (GRα)-mediated functions involved in homeostatic correction, as previously reviewed, ref. [8] and integrates these functions within the Priming, Modulatory, and Restorative phases of critical illness developed in the present Review. The phase-specific assignments incorporate molecular, physiological, translational, and clinical evidence discussed throughout the manuscript. Each phase represents a coordinated set of GRα-mediated responses that are tailored to the host’s evolving physiological demands. The Priming Phase initiates immune activation, metabolic adaptation, antioxidant defense, and cardiovascular responsiveness. The Modulatory Phase focuses on coordinating inflammatory regulation while mitigating oxidative stress, restoring vascular integrity, and preserving systemic homeostasis. The Restorative Phase supports inflammation resolution, tissue repair, adaptive immune recovery, and restoration of normal organ structure and function. This integrated progression enables phase-specific, coordinated, and proportionate adaptive responses to severe physiological stress, promoting the recovery of systemic homeostasis and physiological resilience.

**Figure 3:**
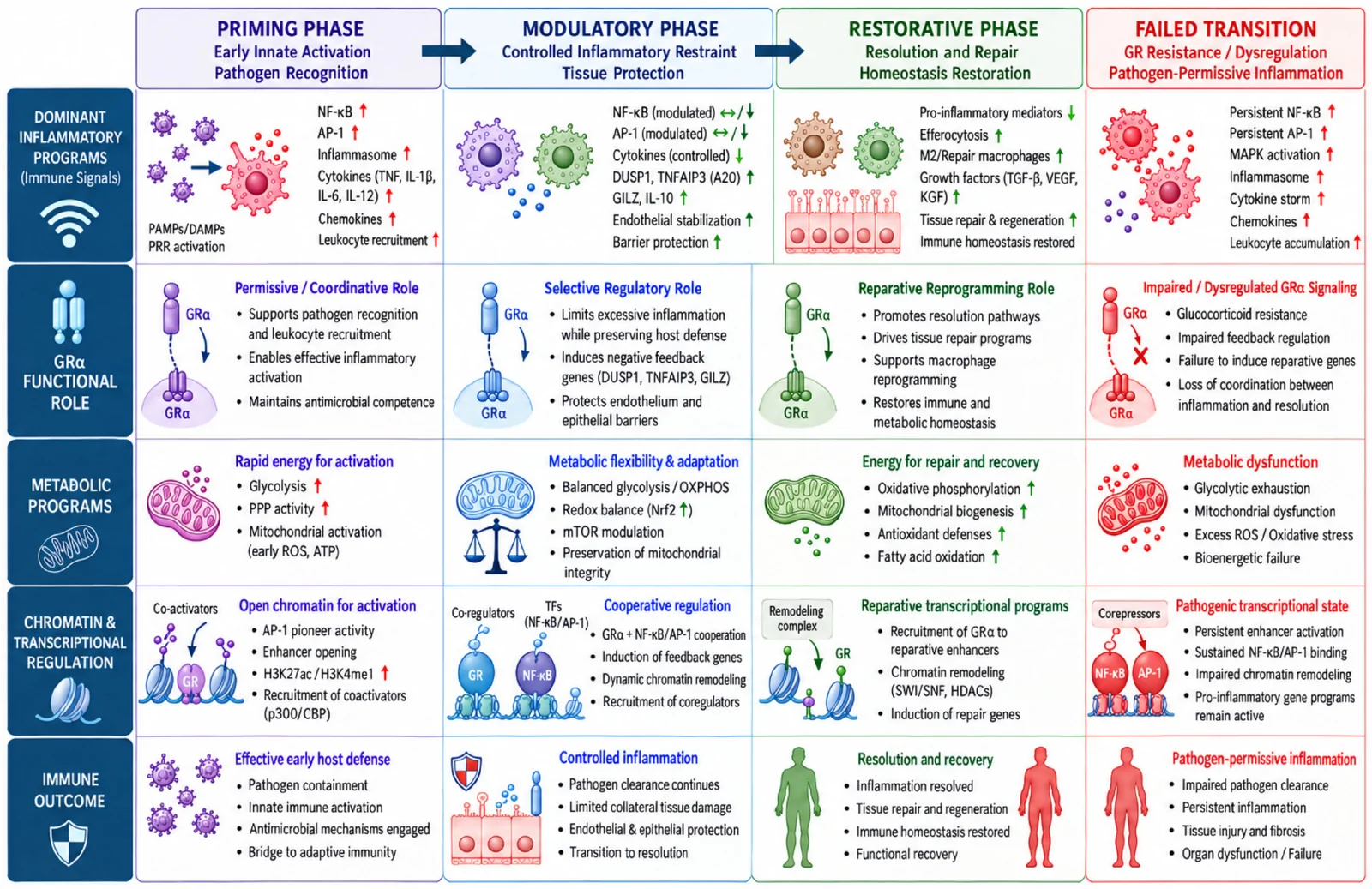
Phase-Specific GRα Regulation of Chromatin Remodeling and Transcriptional Reprogramming During Homeostatic Correction. **Color and arrow coding:** Purple represents the Priming Phase, blue the Modulatory Phase, green the Restorative Phase, and red failed transition or dysregulated responses. Horizontal blue arrows indicate progression between adaptive phases. Upward arrows (↑) indicate increased activity or expression, downward arrows (↓) indicate decreased activity or expression, and bidirectional arrows (↔) indicate maintained or dynamically regulated activity. Red arrows and the red X indicate impaired GRα signaling or failure of normal regulatory responses. Colors are used consistently across the figure to distinguish phase-specific biological processes and outcomes.

The integrated phase-specific regulatory programs through which GRα may support successful homeostatic correction, as well as the consequences of failed phase transitions, are summarized in Figure 3.

This figure illustrates the proposed molecular framework by which glucocorticoid receptor alpha (GRα) contributes to phase-specific changes in chromatin accessibility, transcriptional regulation, and gene-expression programs throughout homeostatic correction. The central GRα regulatory-program column summarizes the evolving phase-specific actions of GRα that coordinate the chromatin remodeling, transcription-factor interactions, and gene-expression changes illustrated in the adjacent columns. During the Priming Phase, GRα promotes adaptive transcriptional activation by facilitating chromatin accessibility, inflammatory gene expression, immunometabolic adaptation, and antimicrobial competence. During the Modulatory Phase, GRα recalibrates inflammatory signaling through coordinated chromatin remodeling, induction of negative-feedback pathways, endothelial protection, and metabolic flexibility. During the Restorative Phase, GRα promotes reparative transcriptional programs that support mitochondrial recovery, inflammatory resolution, extracellular matrix remodeling, tissue repair, and restoration of physiological homeostasis.

The failed-transition pathway illustrates how disruption of coordinated GRα regulatory programs may contribute to persistent inflammatory chromatin states, defective reparative transcription, metabolic dysfunction, glucocorticoid resistance, and failure of homeostatic correction.

This figure emphasizes that successful adaptation reflects dynamic, phase-specific transcriptional reprogramming rather than static inflammatory suppression, thereby providing a molecular framework linking GRα signaling to systems-level homeostatic correction.

The author conceptually developed this figure with assistance from ChatGPT (OpenAI) for graphical generation, visual organization, and figure refinement. The author independently developed, critically reviewed, and approved the biological framework, scientific content, interpretation, and final figure design.

## 7. Limitations and Testable Predictions

The proposed framework has several limitations. Much of the evidence for glucocorticoid–GR signaling derives from cell- and tissue-specific studies, exogenous glucocorticoid exposure, sterile inflammation or injury models, and animal studies that may not fully reproduce human host defense. Moreover, GC–GR responses vary with dose and duration of glucocorticoid exposure, tissue and cell type, receptor abundance and isoform expression, intracellular coregulators, chromatin accessibility, and local glucocorticoid metabolism. The Priming, Modulatory, and Restorative phases should therefore be regarded as overlapping regulatory programs rather than universally validated biological stages, and current evidence does not establish a single GRα-dependent mechanism integrating all biological domains across these phases.

The framework nevertheless generates testable predictions. Longitudinal studies should determine whether shifts among the proposed programs are associated with reproducible changes in immune-inflammatory activity, GC–GR signaling and sensitivity, metabolic and mitochondrial function, vascular integrity, tissue injury, and resolution and repair. It further predicts that these trajectories will differ across tissues and host contexts and that altered tissue-specific GC–GR responsiveness will be associated with transitions among pathogen resistance, disease tolerance, maladaptive host-defense states, and recovery. These predictions can be tested prospectively in experimental infection models and clinically characterized patient cohorts.

## 8. Conclusions

Effective host defense requires far more than eliminating invading pathogens. Successful recovery depends on the coordinated regulation of immune responses and multiple complementary adaptive biological processes that preserve organ function, limit collateral tissue injury, promote disease tolerance, and restore physiological homeostasis. Although these processes have traditionally been studied as largely independent biological systems, growing experimental and clinical evidence indicates that they function as an integrated adaptive network, and that their coordination is essential for successful host defense.

This Review presents a systems-level conceptual framework in which GRα functions as an integrative regulator of host defense by coordinating context-dependent, tissue-specific, and phase-specific adaptive responses throughout homeostatic correction. Rather than attributing previously unrecognized biological functions to GRα, this framework integrates a broad body of established experimental and clinical evidence into a unified, biologically testable model that explains how the diverse actions of GRα collectively support effective host defense. Within this framework, immune regulation is integrated with complementary adaptive biological processes—including endothelial and epithelial barrier integrity, immunometabolic adaptation, mitochondrial bioenergetics, neuroendocrine regulation, lymphatic function, microbiome–host interactions, tissue repair, and inter-organ communication—that collectively enable effective pathogen control, limit immune-mediated tissue injury, and restore physiological homeostasis.

The Priming, Modulatory, and Restorative Phases provide a biologically coherent framework for understanding how these adaptive processes evolve during the host response. Rather than representing independent biological events, these phases constitute a coordinated continuum in which immune activation, inflammatory regulation, metabolic adaptation, tissue protection, and regenerative repair are dynamically integrated according to biological context and the temporal stage of infection. Within this framework, GRα is proposed to contribute to the coordination of transitions among these adaptive programs, thereby supporting both effective antimicrobial defense and the homeostatic correction required for successful recovery.

This systems-level perspective has important implications for future mechanistic investigation and precision medicine. Rather than focusing exclusively on isolated biomarkers or individual signaling pathways, future approaches may benefit from assessing the integrated adaptive capacity of biological systems that collectively determine host defense, disease tolerance, and recovery. Such an approach may improve biological phenotyping, therapeutic monitoring, and the development of individualized interventions to restore adaptive capacity and physiological resilience across acute and chronic disease states.

The principal contribution of this Review is the integration of extensive experimental and clinical evidence into a unified, biologically testable framework for understanding how the diverse actions of GRα may coordinate effective host defense with the complementary adaptive processes required for successful homeostatic correction. If supported by future mechanistic, translational, and clinical studies, this framework may provide a foundation for understanding coordinated host defense and for guiding functional assessment and precision therapeutic strategies aimed at restoring adaptive capacity, physiological resilience, and homeostasis across acute and chronic disease.

## Figures and Tables

**Table 1:** Structured, Hypothesis-Driven AI-Assisted Query Framework for Evaluating GRα Regulation of Immune Sensing, Antimicrobial Defense, and Immune Effectiveness.

Functional Domain	Evidence Domain	Consensus Query	Scientific Objective	Phase of Homeostatic Correction
Pathogen recognition	PRR signaling (TLR, NLR, inflammasome)	Does glucocorticoid receptor alpha (GRα) signaling modulate Toll-like receptor and inflammasome activation, signaling thresholds, and coordinated host-defense responses during infection?	Assess GRα coordination of pathogen recognition, host-defense signaling, and immune effectiveness [10,11,12].	Priming Phase
Innate immunity	Inflammatory signaling	How does GRα coordinate NF-κB– and AP-1–dependent inflammatory and transcriptional programs during host defense and phase-specific homeostatic correction?	Assess GRα regulation of inflammatory signaling and preservation of effective host defense [7,12,13].	Priming Phase
Innate immunity	Antimicrobial function	Does GRα signaling coordinate macrophage antimicrobial competence, phagocytic activity, and intracellular pathogen clearance during host defense?	Assess GRα regulation of antimicrobial competence and pathogen clearance [7,14,15].	Priming Phase
Innate immunity	Immunometabolism	Does GRα signaling coordinate mitochondrial function, immunometabolic adaptation, and bioenergetic regulation during host defense responses?	Assess GRα regulation of immunometabolic adaptation and mitochondrial bioenergetics [16,17,18].	Priming → Modulatory Phase
Inflammation regulation	Cytokine and chemokine regulation	Does GRα regulate the magnitude, duration, coordination, and phase-specific transition of cytokine and chemokine networks during host defense and homeostatic correction?	Assess GRα regulation of cytokine networks during phase-specific host defense [7,13,19].	Modulatory Phase
Host defense outcome	Pathogen clearance vs. persistence	Does GRα help coordinate the transition between pathogen-clearing and pathogen-permissive immune states during infection and homeostatic correction?	Assess GRα regulation of pathogen clearance and recovery-oriented immune responses [3,4,20]	Modulatory → Organ Functional Recovery: Restorative Phase
Resolution and repair	Immune reprogramming and resolution	How does GRα regulate immune reprogramming, resolution pathways, and restoration of immune homeostasis following pathogen clearance?	Assess GRα regulation of inflammatory resolution, tissue repair, and restoration of physiological homeostasis [7,8,21].	Restorative Phase

Abbreviations: GRα, glucocorticoid receptor alpha; PRR, pattern-recognition receptor; TLR, Toll-like receptor; NLR, nucleotide-binding oligomerization domain–like receptor; NF-κB, nuclear factor kappa B; AP-1, activator protein-1.

**Table 2:** Major Functional Domains of Immune Responses Relevant to GRα Regulation.

Immune Response Domain	Principal Components	Primary Function	Relevance to the GRα Conceptual Framework
**Barrier immunity**	Skin, airway, gut epithelium, mucus, antimicrobial peptides, microbiome	Maintains host–environment barrier integrity, regulates microbial interactions, and preserves tissue-integrated host defense	Highlights epithelial and mucosal interfaces as dynamic regulators of microbiome–immune–GRα interactions and host–environment homeostasis [7,8].
**Pathogen recognition and sensing**	Pattern-recognition receptors (TLRs, NLRs, RLRs), inflammasomes	Detects microbial and danger signals and initiates coordinated innate immune signaling and host-defense responses	Highlights the role of GRα in coordinating pathogen-sensing and integrated host-defense signaling programs that shape immune effectiveness and adaptive response outcomes [10,11,12].
**Innate immunity**	Macrophages, neutrophils, dendritic cells, NK cells	Integrates early antimicrobial defense, innate immune signaling, immunometabolic adaptation, and tissue-protective host-defense responses	Defines the role of GRα in coordinating inflammatory signaling, antimicrobial competence, immunometabolic adaptation, and tissue-protective innate host-defense responses across homeostatic phases, antimicrobial competence, immunometabolic adaptation, and phase-specific innate immune responses [7,13,14,15,16,17,18].
**Inflammasome-mediated immunity**	NLRP3 inflammasome, caspase-1, IL-1β, IL-18	Regulates inflammasome-dependent signaling and innate host-defense responses during tissue stress and infection	Highlights the phase-dependent role of GRα in coordinating inflammasome priming, activation, and resolution across host defense and homeostatic correction responses [7,12,13,19].
**Complement-mediated immunity**	Complement proteins, opsonins, membrane attack complex	Supports complement-mediated opsonization, antimicrobial signaling, and inflammatory responses during innate immune defense	Highlights GRα-regulated integration of complement-mediated host defense with inflammatory coordination, tissue protection, and repair responses [7,13].
**Humoral immunity**	B cells, plasma cells, antibodies	Supports antibody-mediated host defense and long-term immune adaptation following microbial exposure	Highlights the role of GRα in regulating B-cell function, antibody responses, and adaptive immune recalibration during host defense and recovery [4,20].
**Cell-mediated immunity**	CD4 T cells, CD8 T cells, NK cells	Regulates adaptive cellular host-defense responses, immune recalibration, and tissue-specific regulation during infection and recovery	Highlights the role of GRα in adaptive host-defense regulation, immune recalibration, and tissue-specific immune responses during infection and recovery [3,4,20].
**Tissue-resident immunity**	Tissue-resident macrophages, memory T cells, local immune cells	Supports tissue-specific immune adaptation, local host-defense responses, and microenvironmental homeostasis within organ systems	Highlights tissue-specific and microenvironment-dependent GRα regulation of local immune adaptation, repair responses, and organ-system homeostasis [8,15,21].
**Systemic immunity**	Circulating leukocytes, antibodies, cytokines, lymphoid organs	Supports systemic immune signaling, host-defense responses, and inter-organ immune integration during physiological stress and recovery	Highlights GRα-mediated integration of immune, endocrine, vascular, metabolic, and circadian regulatory networks involved in systemic host defense and adaptive homeostatic coordination [7,16,17,18].
**Resolution and repair immunity**	Macrophages (M2-like), regulatory T cells, stromal and epithelial cells, pro-resolving mediators	Promotes coordinated inflammatory resolution, tissue repair, and restoration of physiological homeostasis	Directly aligns with GRα-mediated restorative mechanisms involved in inflammation resolution, tissue repair, and return to homeostasis [7,8,21].
**Immune memory**	Memory T cells, memory B cells, long-lived plasma cells	Supports adaptive immune recalibration, long-term host-defense readiness, and maintenance of physiological homeostasis following microbial exposure	Highlights the role of GRα in long-term immune adaptation, coordinated physiological recalibration, and maintenance of systemic homeostasis and resilience following infection [4,8,20].

**Abbreviations**: PRR, pattern-recognition receptor; TLR, Toll-like receptor; NLR, nucleotide-binding oligomerization domain–like receptor; RLR, RIG-I–like receptor; PAMP, pathogen-associated molecular pattern; DAMP, damage-associated molecular pattern; NF-κB, nuclear factor kappa B; AP-1, activator protein-1; IRF, interferon regulatory factor; GRα, glucocorticoid receptor alpha. Note: Bold text identifies the major immune response domains for organizational clarity and does not imply greater biological importance or strength of evidence.

**Table 3:** GRα-Mediated Functions During Homeostatic Correction.

Phase	Key Processes	Functions
**Priming Phase**	HPA Axis Activation and Early GRα-Inducible Transcriptional Response [7,19,35]	**HPA Axis Activation**: Initial ACTH-driven cortisol surge ensures metabolic and immune readiness. **GILZ**: Inhibits NF-κB and AP-1 activity, modulates macrophage responses, and promotes neutrophil clearance, thereby facilitating early immune homeostasis. **DUSP1**: Deactivates MAPKs (ERK, JNK, p38), reducing cytokine production and limiting systemic inflammation; essential for GC anti-inflammatory action in multiple models.
**Priming Phase**	Activating Innate Immunity, Immunometabolic Adaptation, and Cardiovascular Response [10,14,16,17]	**Immune-Cell Mobilization:** GRα supports activation, trafficking, and tissue-directed recruitment of innate and adaptive immune cells involved in early antimicrobial defense and homeostatic adaptation. **Immunometabolic Reprogramming and Bioenergetic Adaptation:** Coordinated mitochondrial biogenesis, glucose utilization, glycolytic activation, and fatty-acid oxidation support host-defense responses, antimicrobial competence, and bioenergetic resilience during early infection. **Innate Immune Coordination and Immune Sensing:** GRα supports acute-phase responses and immune-sensing pathways that regulate antimicrobial defense and inflammatory balance during early infection. **Early Barrier and Mucosal Defense Activation:** GC-GRα supports epithelial defense mechanisms, mucosal immunity, and early containment of microbial invasion. **Transcriptional Coordination and Chromatin Priming:** GRα engages in context-dependent interactions with NF-κB, AP-1, and inflammatory signaling pathways to coordinate chromatin accessibility and adaptive early host-defense responses during homeostatic correction. **Phagocytosis and Pathogen Killing**: GRα-dependent regulation of macrophage responses may support phagocytic function, intracellular pathogen killing, and early antimicrobial defense. **Cardiovascular and Hemodynamic Adaptation**: GRα coordinates adrenergic responsiveness, vascular regulation, and microcirculatory adaptation to preserve tissue perfusion, oxygen delivery, and systemic homeostasis during physiological stress. **Fluid Balance and Hemodynamic Regulation**: GC-GRα interacts with mineralocorticoid receptor signaling to support sodium balance, plasma-volume maintenance, and circulatory stability during physiological stress. **Antioxidant Defense Activation**: Upregulation of SOD, GPx, and catalase to limit oxidative injury during early inflammatory activation.
**Modulatory Phase**	Repressing Inflammation, Mitigating Oxidative Stress, and Restoring Vascular Integrity [7,13,21]	**Regulation of Inflammatory Amplification**: GRα calibrates NF-κB, AP-1, and MAPK signaling to preserve proportional antimicrobial, tissue-protective, and homeostatically adaptive host-defense responses. **Chromatin Remodeling**: GC-GRα increases accessibility to anti-inflammatory genes (MKP-1, IκBα, SGK-1). **Endothelial Protection and Vascular Homeostasis**: GRα coordinates endothelial responses that preserve glycocalyx integrity, microcirculatory stability, tissue-fluid balance, and vascular homeostasis during inflammatory stress. **Vascular Homeostasis and Perfusion Regulatio*****n***: GRα coordinates endothelial and vascular adaptive responses that preserve microcirculatory perfusion, tissue oxygen delivery, and integrated cardiovascular homeostasis during physiological stress. **Preservation of Antimicrobial Competence***:* GRα supports mucosal immunity, neutrophil functional integrity, and host defense mechanisms that help prevent secondary and pathogen-permissive infections. **Barrier and Mucosal Homeostasis**: GRα supports epithelial and mucosal integrity, coordinates host–environment barrier defenses and limits microbial translocation and inflammatory tissue injury. **Redox Regulation and Oxidative Stress Mitigation**: GRα coordinates antioxidant and mitochondrial responses that preserve redox balance and limit oxidative tissue injury during inflammatory stress. **Systemic Homeostatic Integration**: GRα coordinates immune activation, metabolic adaptation, and adaptive bioenergetic regulation to preserve physiological resilience during systemic stress. **Neuroendocrine Integration**: GRα coordinates HPA-axis and autonomic nervous system signaling to integrate stress adaptation, immune–metabolic communication, and maintenance of physiological homeostasis during systemic stress.
**Restorative Phase**	Resolv. ing Inflammation, Facilitating Tissue Repair, Restoring Normal Structure, and Activating Adaptive Immunity [4,8,20,21]	**Coordinated Resolution of Inflammation**: GRα-dependent pro-resolving programs, including Annexin A1, ALXR signaling, and GILZ pathways, support immune recalibration, reparative adaptation, and restoration of tissue homeostasis. **Efferocytosis and Apoptotic Cell Clearance**: Pro-resolving macrophage programs **promote the recognition and clearance of apoptotic cells**, supporting inflammatory resolution, tissue recovery, and restoration of immune homeostasis. **Macrophage Functional Reprogramming: Context**-**dependent expression of pro-resolving, efferocytic, and tissue-reparative macrophage programs that contribute to inflammation resolution and tissue recovery.** **Tissue Repair and Structural Restoration**: GRα-dependent reparative programs support angiogenesis, extracellular matrix remodeling, tissue structural recovery, and restoration of functional integrity during physiological homeostatic recovery. **Barrier and Tissue Restoration:** GC-GRα supports epithelial repair, restoration of tissue barrier integrity, and recovery of mucosal homeostasis. **Extracellular Matrix Remodeling and Fibrosis Regulation**: GRα coordinates reparative remodeling pathways that support tissue restoration while limiting maladaptive fibrotic responses. **Adaptive Immune Recalibration and Memory Formation**: Restoration of adaptive immune coordination supports long-term host-defense readiness, immune memory, and maintenance of physiological homeostasis following recovery. **Neutrophil Clearance**: GILZ expression promotes neutrophil apoptosis, preventing excessive inflammation. **Cellular Homeostasis and Metabolic Recovery**: Restoration of mitochondrial function, redox balance, oxygen utilization, and bioenergetic capacity supports tissue repair and physiological recovery. **Muscle Preservation and Functional Recovery**: GRα coordinates protein metabolic adaptation, muscle integrity, and reparative recovery processes that support physiological resilience and functional reintegration during critical illness. **Organ Functional Recovery**: Restoration of tissue architecture, inter-organ communication, and physiological homeostasis supports organ recovery and long-term resilience following critical illness

**Abbreviations**: ACTH (adrenocorticotropic hormone), AP-1 (activator protein-1), CRH (corticotropin-releasing hormone), DUSP1 (dual specificity phosphatase 1), ERK (extracellular signal-regulated kinase), GC(glucocorticoid), GILZ (glucocorticoid-induced leucine zipper), GR (glucocorticoid receptor), GRα (glucocorticoid receptor alpha isoform), HPA axis (hypothalamic–pituitary–adrenal axis), JNK (c-Jun N-terminal kinase), MAPK (mitogen-activated protein kinase), NF-κB (nuclear factor kappa-light-chain-enhancer of activated B cells).

## Data Availability

No new data were created or analyzed in this study. Data sharing is not applicable.
